# Comparative evaluation over time during mate choice in the green swordtail *Xiphophorus hellerii*

**DOI:** 10.1093/beheco/araf108

**Published:** 2025-09-24

**Authors:** Kathryn Bullough, Bram Kuijper, Laura A Kelley

**Affiliations:** Centre for Ecology and Conservation, University of Exeter, Penryn TR10 9FE, United Kingdom; Centre for Ecology and Conservation, University of Exeter, Penryn TR10 9FE, United Kingdom; Centre for Ecology and Conservation, University of Exeter, Penryn TR10 9FE, United Kingdom

**Keywords:** cognition, methods, perception, sequential choice, sexual selection, signaling

## Abstract

During mate choice, choosers are exposed to a variety of sexual signals varying in both magnitude and the environment in which they are experienced. Previous work assumes that choosers evaluate signal variation from potential mates that are simultaneously viewed and compared. However, this is an extreme scenario, and it is important to also consider sequential presentation of mates, as most animals likely experience both scenarios during mate choice. Using green swordtail fish (*Xiphophorus hellerii*), we assessed whether female preferences for larger males differed when males of different sizes were experienced simultaneously or sequentially. We also investigated the perceptual mechanisms of comparison in both contexts, given recent research suggesting that female preferences are often nonlinear. We found that females consistently preferred larger males, irrespective of whether males were experienced simultaneously or sequentially. However, female preferences were stronger for a male of a given size when viewed under simultaneous, compared with sequential, conditions. During sequential presentation, females compared information from both previously and currently presented males, and interest did not decay with subsequent presentations. Previous research has demonstrated that female green swordtails assessing males simultaneously attend to the relative size difference between males, but we found no evidence of any comparative size assessment. Our study demonstrates that when designing mate choice experiments, it is important to consider how females encounter potential mates in the wild, highlighting that there are clear differences in preferences due to methods of mate presentation and that stochastic adjusting of internal standards of quality frequently occur.

## Teaser

When choosing a mate, female swordtail fish assess their options over both space and time. Using a combination of simultaneous and sequential presentation of males, we show that females use information from both previously and currently shown males to choose their preferred option—usually the larger male. However, their preferences were much stronger when given 2 mates to pick from simultaneously. This shows that females’ preferences are affected by how they encounter potential mates.

## Introduction

Females use comparative evaluation mechanisms when viewing, assessing, and choosing mates that often have a complex combination of male traits (Bateson and Healy 2005). Previous literature assumes that choosers express linear preferences for traits that are larger, brighter, more complex, and more intense (Ryan and Keddy-Hector 1992). Nevertheless, it is now well-established that this is not often the case, with many neurobiological and environmental mechanisms resulting in mate choice being irregular and nonlinear (Ryan et al. 2019). For example, females may perceive and respond to male signals in a discontinuous or proportional manner (Baugh et al. 2008; Akre and Johnsen 2014; Caves et al. 2018; Caves and Kelley 2023), or the costs of taking the time to sample mates may override any preferences the females may have (Milinski and Bakker 1992; Lehmann 2007). All of these factors lead to nonrandom, sometimes nonintuitive mate choices, and ultimately sexual selection (Jennions and Petrie 1997).

In most cases, studies investigating mate choice in animal systems use simultaneous presentation of mates in a 2-choice paradigm, as it is easy to compare stimuli and quantify preferences (Owen et al. 2012). However, this is an extreme scenario of how females encounter potential mates, with animals across a variety of reproductive systems (including leks, harems, solitary species, and social species) likely to experience a combination of both simultaneous and sequential mate presentation in the wild (Jennions and Petrie 1997; Dougherty and Shuker 2015). Using solely simultaneous presentation of stimuli in mate choice experiments could therefore bias mate preference results, and it is important to consider how choices may be influenced under sequential presentation. It has been argued that solely using simultaneous experimental designs can amplify the strength of preferences, as often a dichotomous yes or no response is recorded, even when very minor differences in stimuli are used or when there is only a small difference in association time between stimuli (Wagner 1998; Dougherty 2020). Using sequential mate choice designs in addition to simultaneous designs can be more balanced because they introduce a dimension of risk and cost—the perceived mate encounter rate is lower, and a rejection may lead to reduced or no mating opportunities in the future. A rejection of an option in a sequential test may therefore indicate a stronger or more robust preference (Barry and Kokko 2010; Booksmythe et al. 2011; Dougherty 2020). Indeed, theoretical predictions on the evolution of mate choice and empirical investigations show that the costs of sampling and choosing can have a dramatic effect on a female's mate choice decision, and that experimental design can influence the strength of mate preferences recorded (Real 1990; Dougherty and Shuker 2015).

**Fig. 1.**
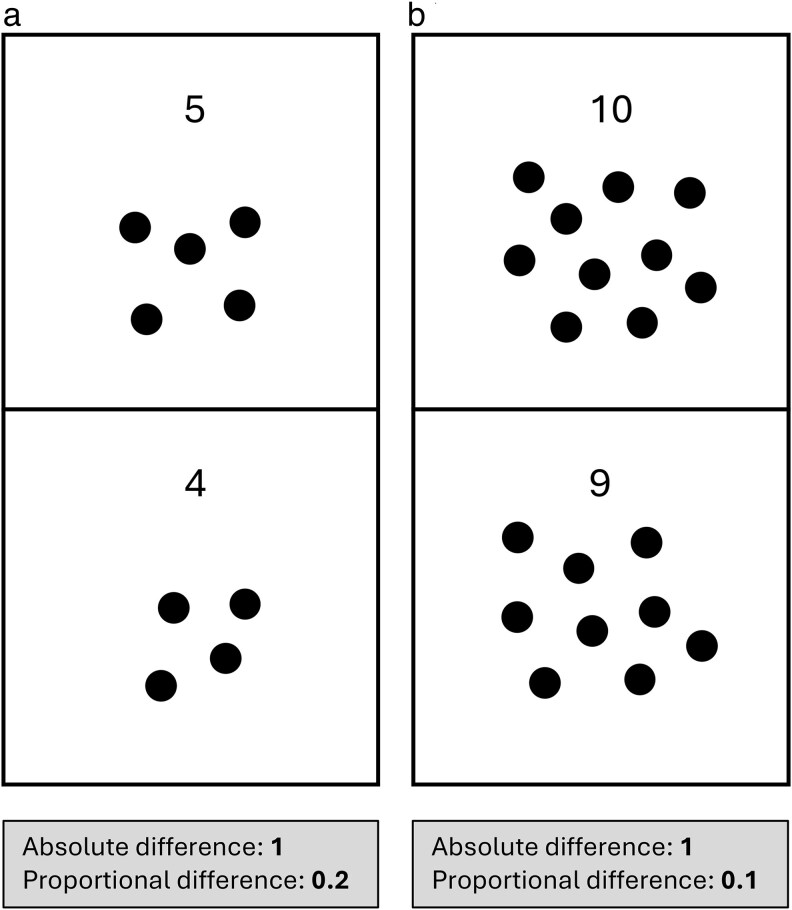
Weber's law of proportional processing states that animals compare the size of stimuli based on their proportional, rather than absolute difference in magnitude. The absolute difference (large—small) in the number of circles in each vertical pair of a) and b) is the same (1), however the proportional difference ([large-small]/large) is greater on a) (1/5 = 0.2) than on b) (1/10 = 0.1), and therefore easier to detect.

Whilst previous work across a range of species with varying reproductive systems (Jennions and Petrie 1997; Dougherty and Shuker 2015; Dougherty 2020) evidently shows that there is an aspect of space and time that females consider when making a mate choice decision, it is currently unclear to what extent the previous stimuli shown has an impact on the choosers' preferences for subsequent stimuli. Sequential mate choice tests by design require that subjects compare a stimulus against either an internal template or to stimuli from memory, so any previous males seen will likely have an impact on whether subjects will accept the new male or wait for future opportunities. It has even been shown that female preferences for specific size-based male phenotypes can be influenced by what males they experience as juveniles (Hebets 2003; Walling et al. 2008). “Previous male effects” have been demonstrated in sticklebacks and guppies, with females following stochastic decision rules attuned to the attractiveness of present and previously encountered males (Bakker and Milinski 1991; Pitcher et al. 2003). However, the specific perceptual and cognitive mechanisms of how females assess and compare these males over time have not been investigated. When presented with male stimuli simultaneously, it has been documented in multiple species that comparisons often occur using the relative (or proportional) difference between stimuli rather than the absolute difference. This is known as Weber's Law of proportional processing, where animals compare the size of stimuli based on their proportional (or relative) difference in magnitude, rather than absolute difference in magnitude (Fig. 1; Bullough et al. 2023; Caves and Kelley 2023; Akre et al. 2011). For example, Caves and Kelley (2023) demonstrated in swordtail fish *Xiphophorus hellerii* that females had stronger preferences for larger males dependent on the proportional, rather than absolute, difference in body sizes of males presented simultaneously, because larger proportional differences are easier to detect. It is yet to be determined whether this mechanism of comparison also occurs when males are presented sequentially rather than simultaneously, or whether female decisions are affected by males they have recently experienced.

In this study, we aimed to investigate whether experiencing potential mates simultaneously in pairs or sequentially individually affected female preferences for larger males in green swordtail fish (*X. hellerii*). In this species, females are likely to encounter males both simultaneously and sequentially: males establish dominance hierarchies and compete for access to choosy females who prefer males with larger body sizes (MacLaren 2017), a heritable trait which is linked to mating success and fitness (Campton 1992). Swordtails respond well to virtual animated stimuli, meaning stimulus traits can be finely controlled, and fish in captivity exhibit normal courtship and mate choice behavior (Trainor and Basolo 2000; Walling et al. 2010; Caves and Kelley 2023). We had 2 distinct aims: comparing the strength of female preference (in terms of association time) for larger males in both simultaneous and sequential mate choice designs, and building on work carried out by Caves and Kelley (2023) to investigate the perceptual mechanisms underlying comparisons of male size. We predicted that female swordtails would have stronger preferences for larger males in the simultaneous experiments, as they could more easily compare and evaluate which male is bigger. In contrast, in the sequential experiments, we predicted preferences for larger males would be weaker, as without a direct comparison between males, females necessarily rely on previous experiences when expressing preferences. Finally, we expected that comparisons of both simultaneous and sequential stimuli would occur based on relative size differences, rather than absolute differences, consistent with Weber's Law. Consequently, we predicted that during simultaneous assessment, females would exhibit stronger preferences for the larger male in a pair when assessing males that were generally smaller (a large proportional difference) and weaker preferences for the larger male in a pair when assessing males that were generally larger (a smaller proportional difference). In sequential trials, we expected that the strength of female preference for the current male would reflect the proportional size difference between the current and previously viewed male.

## Materials and methods

### Animal care and ethics

We used adult green swordtails that were descendants of a population collected from Mexico, although the exact collection location and date were unknown. Fish were bought into the aquatic facility in April 2022 and all fish used in these experiments were bred on site. All animals were treated in accordance with the ethical guidelines of the University of Exeter (ethics approval number 488337). Fish handling and experiments were carried out by KB (Home Office Personal Licence I42806858) under Home Office Project Licence PF6E68517. Fish were housed in single-sex groups of 15 to 30 individuals in 30-L tanks, where the water temperature was kept between 22 and 24 °C. Tanks were lit from above with AquaBeam LED lights (Tropical Marine Centre Ltd.) on a 12:12 light:dark cycle. All fish were tagged by LAK (PIL I92638227) under anesthesia with an individually identifiable combination of elastomer tags (Northwest Marine Technology Inc.).

### Stimulus design

Artificial animated stimuli (to standardize phenotype and allow for precise size scaling) comprising of a digital image of a male green swordtail were scaled either up or down to create 6 male stimuli varying in body size area—120% (1,054 mm^2^), 110% (885 mm^2^), 100% (721 mm^2^), 97% (692 mm^2^), 85% (533 mm^2^), and 70% (363 mm^2^). The 100% stimulus (body length 44 mm, sword length 32 mm) matched the mean overall body area of the housed population of males, and the range of stimulus sizes presented were within the natural range of variation in this species when displayed during trials on a Samsung Galaxy Table 10.1 tablet (Samsung Corp; 22.3 × 14 cm screen, 1,200 × 1,900 pixel resolution, 60 Hz refresh rate; Caves and Kelley 2023). An artificial female dummy stimulus (to control for possible schooling effects in the sequential trials; MacLaren and Rowland 2006) was created using a digital image of a female swordtail taken from the experimental population and scaled to match the mean overall body length of the experimental population (53 mm) when displayed on the same tablets.

Animations were then created using Microsoft PowerPoint (as detailed in Caves and Kelley 2023). Animation paths were used so that the male stimulus moved from one side of the screen to the other in 15 s, left the screen, and then reappeared moving in the opposite direction for a further 15 s. We also included 3 backwards swim maneuvers every 60 s to imitate the movements of a courting male. For the female stimulus, animation paths were used to animate the stimulus moving from one side of the screen to the other over 15 s to match the duration that the male stimuli were on-screen, without the backward-swim courting maneuvers.

### Preparation of trials

Females (*n* = 30) were housed together to be physically and visually isolated from males as soon as juvenile sex determination was possible. The same 30 females were used in both the simultaneous and sequential experiments, with a minimum 3-wk duration between experiments. For logistical reasons, we conducted experimental trials in parallel by testing 2 females simultaneously in separate experimental tanks. Immediately prior to a trial, 2 females were caught using a dip-net and placed into the middle of a tank containing a group of males (*n* = 9) on either side for 15 min, placing the females in visual, but not physical contact with the males. Each female was then moved to an individual 2-choice test tank (45.7 × 25.4 × 25.4 cm), filled to a depth of 15 cm using water from the home tank system. Tanks were filmed from above using a Sunkwang C160 video camera with 6 to 60 mm manual focus lens suspended above the tank and connected to a computer running the Viewer tracking software (BiObserve). The software virtually divided the tank into thirds, and tracked all movements made by the fish for the duration of the trial. Each third at the ends of the tank were considered “choice zones,” where time spent in these thirds closest to the stimulus was used as a measure of preference for that stimulus. A piece of black tubing was provided in the center third as shelter. A cardboard screen was placed around the tank prior to the trial to prevent external visual disturbance.

### Simultaneous 2-choice trials

Experimental protocols from Caves and Kelley (2023) were followed to carry out the simultaneous mate choice trials. Individual females prepared for trial as described above were placed inside of a clear acrylic cylinder (15 cm diameter) in the center third of their individual 2-choice test tank for 12 min prior to the start of the trial. During this time, 2 display tablets were placed against each end of the tank, displaying a plain gray background for 11 min for acclimation, followed by 1 min of a male stimulus, allowing the female to view both male stimuli before being allowed to make a decision. During this acclimation period, a small amount of water from a tank housing male swordtails was added to the arena, providing the female with olfactory cues to prime her for mate choice. The cylinder was then removed, and the female was allowed to access the entire tank whilst the male stimuli continued to play at each end for 3 min, during which time all movements by the female were tracked and recorded by the Viewer software. Following trials, the female was returned to her home tank and Viewer was used to extract and record the amount of time the female spent in each zone. Forty-eight hours after presentation of 1 set of stimuli, the same procedure was followed with the same female and the same pair of male stimuli, but with each stimulus male presented on the opposite side of the tank to the first trial to account for possible side biases.

A total of 7 pairs of male stimuli were presented to each female in random order: 120% vs. 70%, 120% vs. 85%, 120% vs. 110%, 120% vs. 100%, 110% vs. 70%, 97% vs. 70%, and 85% vs. 70%, with all pairs being presented over the course of 7 wk. The pairs were selected to cover a range of both proportional and absolute differences, and so that 3 pairings of stimulus pairs were created in which the absolute difference was the same, but the proportional difference was not. For example, we created 2 sets of stimulus pairs in which the absolute difference was 170 mm^2^, but in one of the pairs, proportional difference was 0.32 (85% vs. 70%) and in the other, it was 0.16 (120% vs. 110%). This allowed us to directly test the effects of proportional difference on preference while holding absolute difference constant. Within individuals, the side that the larger male was presented on in the first trial was randomized for each set of stimulus pairs, and all females were shown the stimulus pairs in the same order, as presentation order was previously found to not affect female preferences (Caves and Kelley 2023). In total, 30 females were used, with 2 trials per stimulus pair, for a total of 420 trials. Sixteen of these simultaneous trials were removed from analysis due to errors in following the experimental protocol (failure of PowerPoints to run, Viewer inaccurately tracking the fish's location, failure of Excel to extract Viewer data).

### Sequential 2-choice trials

Following simultaneous presentation of male stimuli, after a minimum of 2 wk in their home tanks, the same stimuli were presented to female swordtails in a sequential manner to determine if this would impact their strength of preferences and method of size comparison.

Individual females prepared for trial as described above were placed inside of the clear acrylic cylinder in the center third of their individual 2-choice test tank for 12 min prior to the start of the trial. During this time, 2 display tablets were placed against each end of the tank, displaying a plain gray background for 11 min for acclimation, followed by 1 min of a male stimulus on one side and the dummy female stimulus on the other (to control for possible schooling effects), allowing the female to view both stimuli before being allowed to make a decision. During the acclimation, a small amount of water from the male tank was added to the arena.

The cylinder was then removed, and the female was allowed to access the entire tank whilst the stimuli continued to play at each end for 3 min. The female was then gently moved back into the clear acrylic cylinder in the center third of the tank, following which another acclimation period was undertaken. This consisted of a further 2 min of plain gray backgrounds, followed by 1 min of male stimulus showed on the same side of the tank as previously and the female stimulus on the other. Again, during this acclimation period, a small amount of water from the male tank was added to the arena. The cylinder was then removed again, and the female was allowed to access the entire tank whilst the stimuli continued to play at each end for 3 min. This protocol was repeated until the female had seen all 6 male stimuli in a sequential order on the same side of the tank. Following trials, the female was returned to her home tank and we recorded the amount of time the female spent in each zone during each 3 min “trial period.”

The same male stimuli as in the previous experiment were used: 120%, 110%, 100%, 97%, 85% and 70%, and the order of presentation of male stimuli was randomized for each female. Two weeks after presentation of 1 sequence of stimuli, the same procedure was followed with the same female and the same order of male stimuli, but with each stimulus presented on the opposite side of the tank to the first trial to account for possible side biases.

In total, 30 females were used, with 2 sides of presentation of the 6 male stimuli per female, for a total of 360 trials. None of the sequential trials was removed from analysis.

### Statistical analyses

For both experiments, any trials where the female fish did not leave the central zone of the tank were removed to ensure that only trials where the female was interacting with the stimuli were analyzed. This meant that for the sequential experiment, 3 fish over 6 trials were removed from analysis. No fish were removed from analysis for the simultaneous experiment. For both experiments, the time spent with each male stimulus was averaged over the 2 repeated trials, to create the response variable used in analyses.

#### Female preferences in simultaneous trials

For the simultaneous 2-choice trials, we used generalized linear mixed-effects models, with size difference between males as a fixed effect and female fish ID as a random intercept effect to determine whether male stimulus body area impacted the amount of time females spent with each male stimulus. We used a Gaussian family error structure, and all models went through diagnostic checks, including checks for overdispersion and residual homogeneity. To investigate the mechanisms females use to compare males simultaneously, the difference in male size was calculated in 2 ways: the absolute difference (largest male size—smallest male size) and the proportional difference ([largest-smallest]/largest). Because absolute and proportional differences in male body size are highly correlated (Pearson correlation coefficient, *r*_5_ = 0.91, *P* = 0.005), we could not include both terms in the same model. We used a model comparison approach based on the Akaike information criterion for small sample sizes (AICc) to determine which assessment strategy best described female preferences (Akaike 1974; Burnham and Anderson 2002; Burnham et al. 2011). We built 4 models to determine whether the difference in body size of the 2 stimuli shown had an impact on the time females spent with each stimulus—an absolute model, using the absolute difference in body area between the 2 males, a proportional model, using the proportional difference between the 2 males, a “mean” model, using the mean body size of the 2 males shown (the rationale being that greater overall stimulation of a female's visual system might weaken preferences for the larger male), and a null model with no fixed effects. In all models, focal female fish ID was included as a random intercept effect.

#### Female preferences in sequential trials

For the sequential 2-choice trials, we used generalized linear mixed-effects models with female fish ID as a random intercept effect, to determine whether male stimulus body area and order of presentation impacted the amount and proportion of time that females spent with each male stimulus. We used a Gaussian family error structure, and all models went through diagnostic checks, including checks for overdispersion and residual homogeneity. To investigate the mechanisms by which females compare males sequentially, AICc scores were used to determine whether the current male body area or body area of previous male stimuli had an impact on the time females spent with the current male stimulus. Five models were built to be compared for best fit—a “current male” model, where only the body size of the current male was included, a “previous absolute” model, using the absolute difference in body area between the current and previous male, a “previous proportional” model, using the proportional difference in body area between the current and previous male, an “average previous absolute” model, using the absolute body area difference between the current male and the running body size average of all previous males seen, and a null model without any fixed effects. In all models, focal female fish ID was included as a random intercept effect. Repeatability of the time females spent with each male stimulus across the 2 repeated experiments was examined by calculating the intra-class correlation (ICC), as part of the “rptR” package.

#### General preference: simultaneous vs. sequential

To compare female preferences for males of each size between the simultaneous and sequential 2-choice trials, the data were condensed down so that each individual female fish had an average time spent with each male stimulus over all of their experiments for each trial type. A paired *t*-test and a mixed-effects model, including female fish ID as a random effect were then used to compare the average time spent with each stimulus for both simultaneous and sequential trials.

All analyses were carried out using RStudio version 4.3.0 including packages “dplyr 1.1.4,” “tidyr” 1.3.1, “ggplot2” 3.5.1, “Rmisc” 1.5.1, “lme4” 1.35.5, “lmerTest” 3.3, “ggpubr” 0.6.0, “rptR” 0.9.22, and “MuMIn” 1.48.4.

## Results

### Simultaneous presentation

**Fig. 2.**
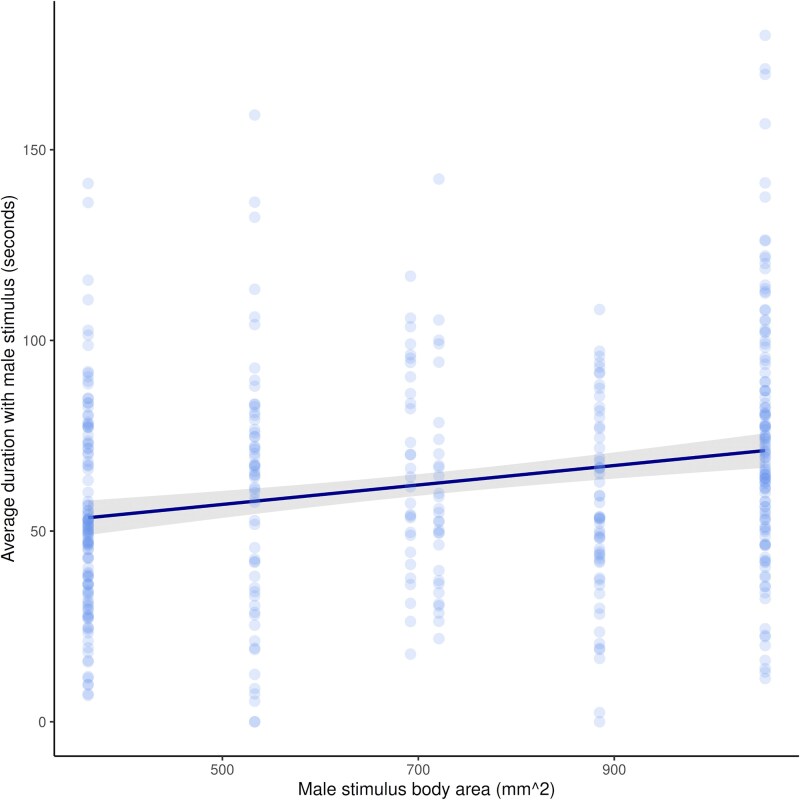
Females (*n* = 30) spent more time on average with male stimuli that had a larger body area (linear mixed-effects model with a response variable of association time, predictor variable of male body area, and female fish ID as a random effect). Points represent average over 2 trials for each female fish shown each stimuli comparison, and the trendline shows model estimates with a 95% confidence interval.

**Fig. 3.**
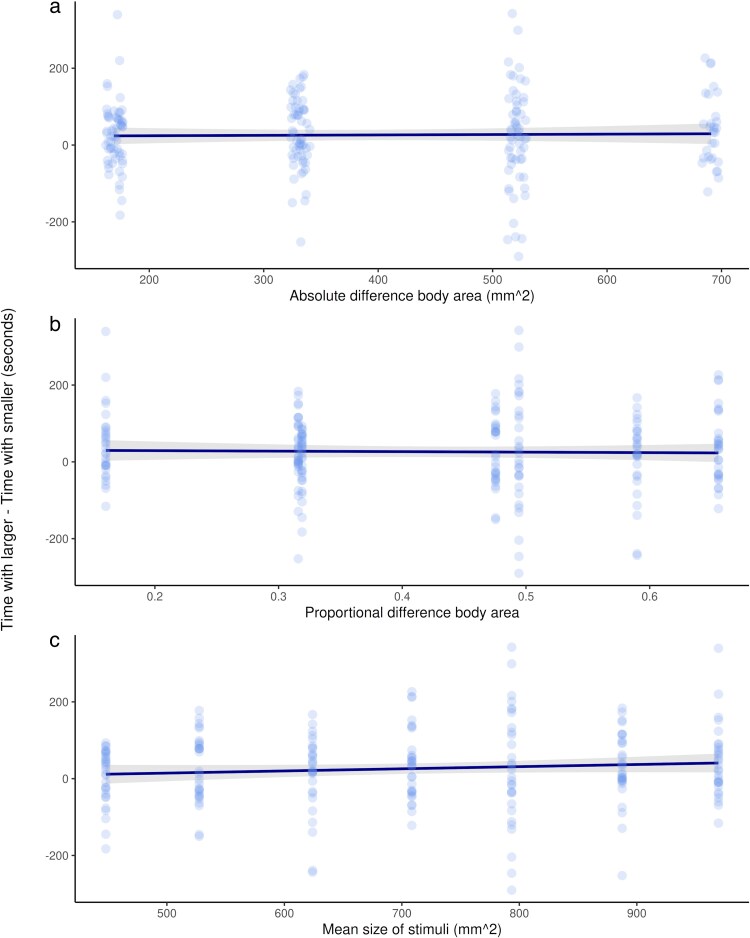
Females (*n* = 30) do not compare male body size areas of male stimuli based on a) the absolute difference (predictor variable of the absolute difference between the body area of the 2 males displayed), b), the proportional difference (predictor variable of the proportional difference between the body area of the 2 males displayed), or c) the mean size (predictor variable of the mean body area of the 2 males displayed) of the stimuli shown when presented simultaneously (linear mixed-effects models with a response variable of association time and female fish ID as a random effect). Points represent average over 2 trials for each female fish shown each stimuli comparison, and the trendline shows model estimates with a 95% confidence interval.

Overall, females spent a significantly greater amount of time with male stimuli that had a larger body area (mixed-effects model; *β*_area_ = 0.03*x* + 43.28, *χ*^2^ = 22.03, df = 1, *P* < 0.001; Fig. 2). Despite this, there was no model that overwhelmingly had the best fit overall. Whilst the null model was the best fit, the mean, proportional, and absolute models were all within 2AICc (Fig. 3, Table 1), suggesting that females may not compare male body size areas of male stimuli based on the absolute difference, the proportional difference, or the mean size of the stimuli shown when male stimuli are presented simultaneously.

**Table 1.** Summary of model response and predictor variables, *k* values (number of fitted parameters), ΔAICc and log-likelihood values, and model weights (*w_i_*) for simultaneous mate presentation trials.

Model	*k*	Log likelihood	ΔAICc	*w_i_*
Preference ∼ 1 + (1|Fish ID)	3	−1,254.03	0.00	0.368
Preference ∼ mean difference + (1|Fish ID)	4	−1,253.01	0.04	0.360
Preference ∼ relative difference + (1|Fish ID)	4	−1,253.98	1.99	0.136
Preference ∼ absolute difference + (1|Fish ID)	4	−1,253.99	2.00	0.135

For each model set, the best-fit model is listed first.

### Sequential presentation

**Fig. 4.**
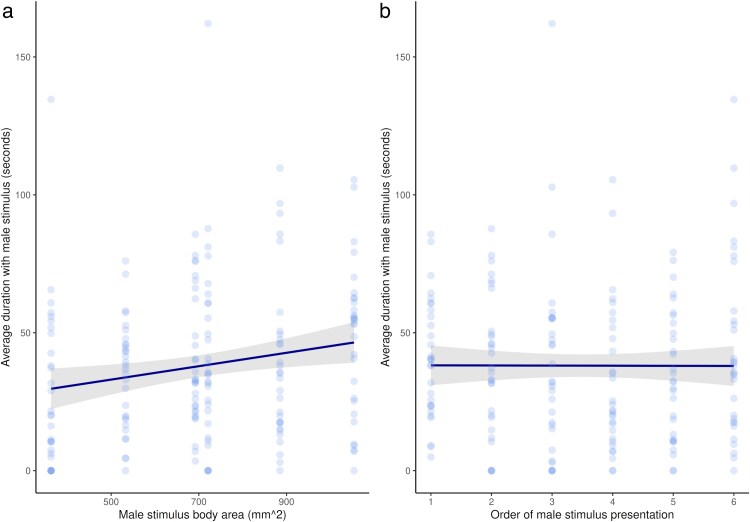
Females (*n* = 30) spend a) more time on average with male stimuli that have a larger body area (linear mixed-effects model with a response variable of association time, predictor variable of male body area, and female fish ID as a random effect), but b) do not change the average time spent with male stimuli based on the order they are presented in (linear mixed-effects model with a response variable of association time, predictor variable of order of stimulus presentation, and female fish ID as a random effect). Points represent average over 2 trials for each female fish shown each stimuli size, and the trendline shows model estimates with a 95% confidence interval.

Overall, females spent the majority of time associating with the dummy female (64.69 ± 33.26 s, mean ± SD) rather than the male stimulus (38.07 ± 27.84 s; the remaining time was spent in the center third of the tank), with focal females preferring the male stimulus over the dummy female in only 31% of trials. Despite this, females tended to prefer males with a larger body area, spending a greater percentage of their time with these larger stimuli when compared with the dummy female (mixed-effects model; *β*_area_ = 0.62*x*–86.82, *χ*^2^ = 9.25, df = 1, *P* = 0.002; Fig. 4a). There was no effect of order of presentation on the time females spent with the male stimuli (mixed-effects model; *β*_order_ = −0.04*x* + 38.23, *χ*^2^ = 0.002, df = 1, *P* = 0.97; Fig. 4b). Repeatability of the time females spent with each male stimulus across the 2 repeated experiments was low (ICC = 0.136 [confidence interval (CI): 0.045, 0.234]).

**Fig. 5.**
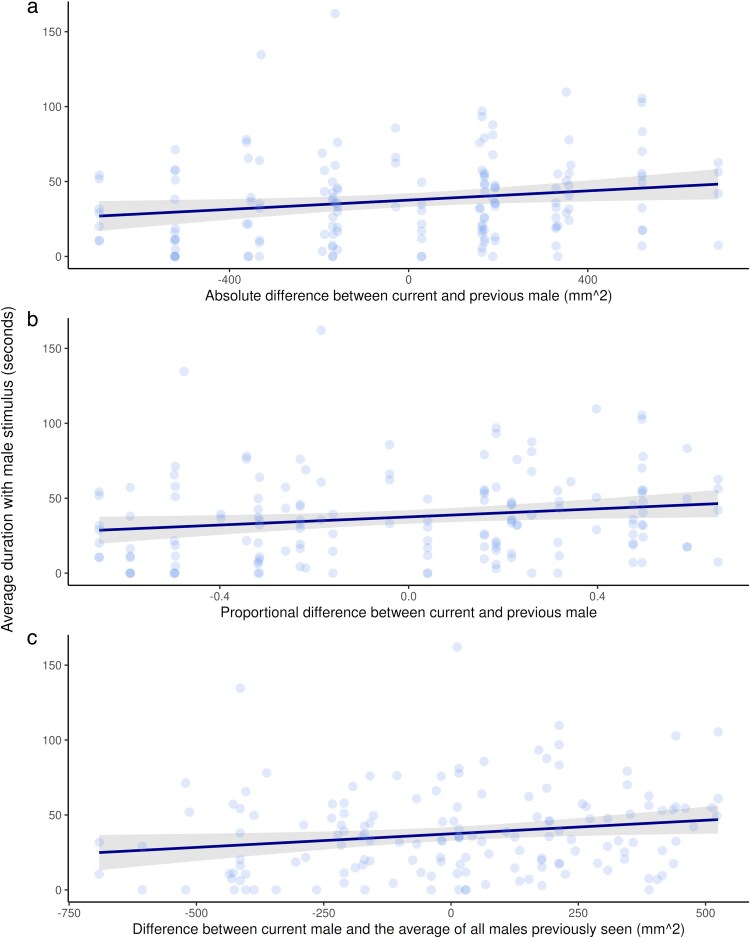
Females (*n* = 30) spend more time on average with the current male stimuli when the a) absolute difference (predictor variable of the absolute difference between the body area of the current and previous males), and b) proportional difference (predictor variable of the proportional difference between the body area of the current and previous males) between the body area of the current male stimuli and the previous male stimuli shown is larger (linear mixed-effects models with a response variable of association time and female fish ID as a random effect). c) They also spend more time on average with the current male stimuli when the difference between the body area of the current male stimuli and the running average of all previous males seen is larger (the predictor variable, linear mixed effects model with a response variable of association time and female fish ID as a random effect). Points represent averaged trials for each female fish shown each stimuli size, and the trendline shows model estimates with a 95% confidence interval.

When evaluating how females responded based on which previous male stimuli they had seen, there was no model that overwhelmingly had the best fit overall. Whilst the previous absolute model was the best-fit model (Fig. 5a; Table 2), the proportional, average previous, and current male models were all within 2AICc (Figs. 4a, 5b and c, Table 2). The model taking into account only previous male area and the null model were worse fits (Table 2).

**Table 2.** Summary of model response and predictor variables, *k* values (number of fitted parameters), ΔAICc and log-likelihood values, and model weights (*w_i_*) for sequential mate presentation trials.

Model	*k*	Log likelihood	ΔAICc	*w_i_*
Preference ∼ absolute difference + (1|Fish ID)	4	−702.91	0.00	0.341
Preference ∼ proportional difference + (1|Fish ID)	4	−703.34	0.85	0.223
Preference ∼ average difference + (1|Fish ID)	4	−703.45	1.06	0.200
Preference ∼ current male size + (1|Fish ID)	4	−703.56	1.30	0.178
Preference ∼ previous male size + (1|Fish ID)	4	−705.17	4.52	0.036
Preference ∼ 1 + (1|Fish ID)	3	−706.72	5.51	0.022

For each model set, the best-fit model is listed first.

### Comparing simultaneous and sequential presentation

**Fig. 6.**
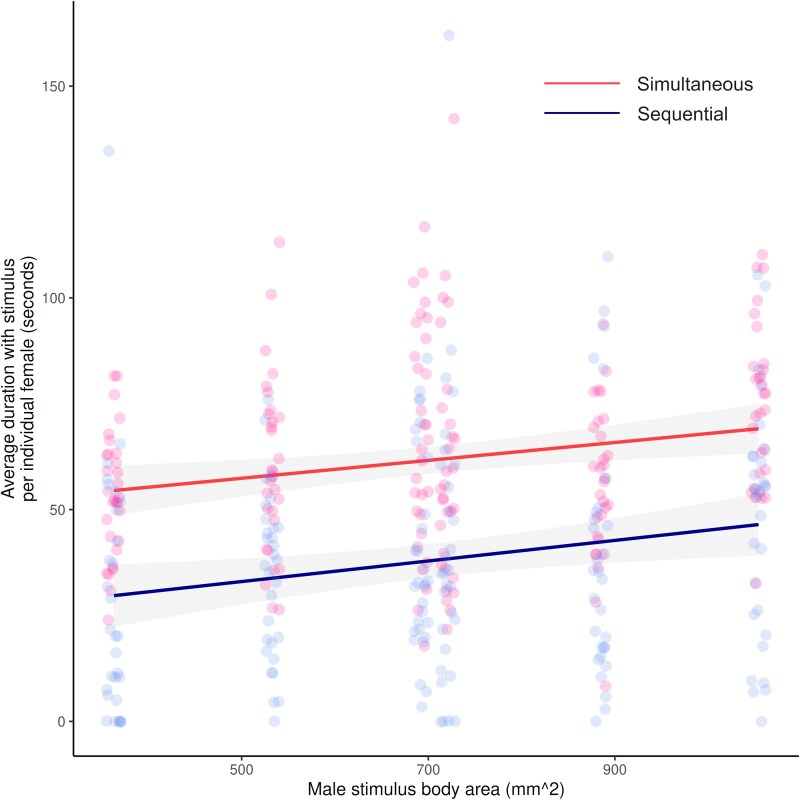
Females (*n* = 30) spend more time on average with male stimuli that have a larger body area in both simultaneous (red) and sequential (blue) 2-choice trials (linear mixed-effects model with a response variable of association time, predictor variables of male body area and type of trial, and female fish ID as a random effect). However, the strength of this preference is significantly higher in the simultaneous trials. Points represent averaged trials for each female fish shown each stimuli size, and the trendline shows model estimates with a 95% confidence interval.

When comparing both the simultaneous and sequential 2-choice trials, the way in which males were presented to females affected the strength of female preference for the male stimuli, with simultaneous trials eliciting a greater time spent with each male stimulus (paired *t*-test; *t* = 9.64, df = 179, *P* < 0.001; Fig. 6). This result was equivalent to the associated mixed-effects model (*β*_trial type_ = 23.73*x* + 38.07, *χ*^2^ = 79.83, df = 1, *P* < 0.001). However, there was no interaction between the type of trial used and the body area of the male stimuli (mixed-effects model; *β*_interaction_ = −0.003*x* + 20.87, *χ*^2^ = 0.08, df = 1, *P* = 0.77; Fig. 6), meaning the type of trial did not affect general female size preferences overall, just the strength of that preference.

## Discussion

Overall, although female swordtails preferred larger male stimuli in both the simultaneous and sequential mate presentation designs, for both experiments there was no one model that ultimately explained how they were comparing them. Models suggested that there was some form of comparison of stimuli occurring in the sequential experiment, which included comparisons of the previous male with the current male, but it is unclear whether these comparisons were absolute, proportional, or involved a mean value of the previous stimuli seen. Despite this, it was evident from the increased duration of time spent with the preferred male that simultaneous presentation of stimuli resulted in stronger preferences overall for each male stimulus.

When male stimuli were presented sequentially, females seemed to take into account both the current male and the previous male when making a mate choice decision. This is evident from the increased duration of time spent with the preferred males when the current male was larger and had a greater size difference compared with previous males. Several rules have been suggested according to which females decide on mates encountered sequentially. Females may choose the first male encountered that meets a minimum quality criterium (fixed threshold rule; Janetos and Cole 1981), may mate with any male exceeding the criterium (threshold criterium rule; Wittenberger 1983), or may continually compare males sequentially and mate when the quality encountered is greater than the quality expected from continued search (1-step decision and adjustable threshold rules; Janetos and Cole 1981; Reid and Stamps 1997). The optimum way of sampling males sequentially is according to the “best of n rule,” where females sample as many potential mates as possible and then mate with the highest quality male (Janetos 1980), although once search costs are taken into account, a fixed threshold becomes the optimal way to sample males sequentially (Real 1990). In our study, the female could not access any previous males and were primed for this possibility of mate deprivation due to being kept in single-sex housing, making mate rejection costly.

Our results suggest that, in reality, mate sampling and choice are unlikely to be as simple as the models we fitted—as there were no order effects found, it appears that either all the males were above the ideal threshold, or that the threshold does not exist (within biological reason). The females in this experiment had been isolated from males as soon as they could be reliably sexed after birth (to control mate exposure and experience across females and to avoid pregnancies), so their thresholds may have been disrupted due to mate deprivation. In the wild, females would typically encounter males regularly in both space and time, so it is possible that isolation may have impacted their mate preferences and thresholds. However, female preferences were not random, suggesting that some form of memory-based comparison and attuning of behavior to male quality was occurring. It is more likely that some form of flexible adjusting of the internal standard of quality is happening, where if the difference between the males seen is larger, the female is more likely to mate with the current male (Bakker and Milinski 1991). This contrasts with other studies examining sequential presentation in guppies, where females were relatively indiscriminate at first, then became increasingly choosy with each successive mating to “trade up” (Pitcher et al. 2003). This difference between our study and Pitcher et al. (2003) may arise because female swordtails encounter males who have clear dominance hierarchies and cues of quality, so they can afford to be more choosy from the outset compared with guppies. Another possible factor is that our study used videos of males, meaning that females did not change reproductive status with successive encounters, in contrast to actual males that were allowed to copulate with assessing females in Pitcher et al. (2003). Precopulatory mate choice in swordtails may be very stochastic as once inseminated, *Xiphophorus* species can store sperm for up to a year (Constantz et al. 1989; Potter and Kramer 2000; Huang et al. 2004), meaning that postcopulatory sexual selection may play an important role in this group of fishes (Simmons et al. 2008; Gasparini et al. 2010; Smith 2012), although postcopulatory mate choice has not yet been examined empirically in this species. It would be valuable to look at whether males encountered even further back than the immediately previous male, or whether allowing females to actually mate with real males, impacts female preferences.

One of the key ideas we wanted to investigate in this study was the underlying mechanisms of how females compare different magnitudes of stimuli over a temporal context. It has been previously shown that responses to simultaneous presentation of stimuli are not linear (Baugh et al. 2008; Akre and Johnsen 2014; Caves et al. 2018; Caves and Kelley 2023), with the simultaneous experiment in this study being a replica of the study by Caves and Kelley (2023) demonstrating Weber's Law of proportional processing during mate choice in this species. It is interesting that we did not find evidence of proportional processing, when the only aspect changed was the specific population of individual swordtails used: the swordtails used in this study were collected from Mexico, while the population in the study from Caves and Kelley (2023) was collected from Belize. Our findings suggest that females in this study may be less sensitive to the social context that males are presented in, in contrast to the findings of Caves and Kelley (2023), where females clearly compared males based on their relative size differences. This may be due to a variety of reasons—population-specific differences due to genetic drift, local adaptation to different environments, long-term social rearing environment in captivity, and the extent of inbreeding may all play a role in the cognitive mechanisms and motivations that females use to choose mates. We also did not find any strong evidence of a proportional response in the sequential part of the experiment, even though it has been demonstrated that Weber's Law can have a temporal application (Namboodiri et al. 2014; Gomez-Laplaza and Gerlai 2016), has been modeled in temporal risky choice and foraging scenarios (Kacelnik and e Abreu 1998), and that *Xiphophorus* fish likely have sufficient memory for it to apply (Chance 2008; Queller et al. 2023).

Females had weaker preferences for male body sizes when they were presented sequentially rather than simultaneously. In terms of cognition, this likely happens because it is much easier to compare and assess differences between stimuli when they are both visually present in the simultaneous trials, resulting in much quicker and stronger decision-making (Bateson and Healy 2005; Beatty and Franks 2012; Dougherty and Shuker 2015). In terms of perceived risk, it may be because the cost of rejecting an option in the simultaneous experiment is zero, whilst in the sequential experiment it is much higher as the subject does not know if it will get another chance to mate, resulting in lower exhibited preferences and more random mating (MacLaren and Rowland 2006; Dougherty and Shuker 2015). However, Dougherty and Shuker (2015) suggest that there should be no difference in the strength of preference between paradigms once the perceived mate encounter rate is controlled for, such as by giving subjects experience of the same number of mates before testing. In our experiment, all females had identical exposure to males before being isolated for the mate choice experiments, so the results seen here may not be due to perceived risk. In addition to this, in the wild, green swordtails would be unlikely to run out of mating options due to their relatively population-dense habitats. Combined with the fact that their reproductive cycles are relatively quick and that females have low reproductive costs compared with other taxa (such as mammals with limited offspring), it is doubtful that they undergo much risk by rejecting a mate in the wild.

It has also been suggested that sampling strategy can dramatically influence the female preferences recorded in mate choice experiments, as even if females have identical preferences, they may sample males differently depending on the options available in the trial arena (Wagner 1998; MacLaren and Rowland 2006). For example, females may sample stimuli repeatedly before making a choice, some may sample each stimulus only once before choosing, or some may remain in a “safe zone,” assessing each option before choosing. In our sequential experiment, females spent most of their time associating with the dummy female—if this is their “safe zone” to remain in before choosing, it may explain why preferences in the sequential experiment were much lower than in the simultaneous experiment, where they had males on either side. It also may highlight an issue that if females are overwhelmingly choosing to spend time with other females, they may not be interested in mating at all and that their decisions are motivated solely by safety. This can be explicitly tested in sequentially designed mate choice experiments, unlike simultaneous experiments that typically offer a binary choice between males with no female option. Either way, this result clearly highlights how the type of mate encounter can impact female preferences. In the wild, swordtails likely experience both simultaneous and sequential mate choice, as individual populations are highly mixed with multiple matings occurring for both females and males (Beaugrand et al. 1984; Simmons et al. 2008; Smith 2014). When designing mate choice experiments, considering how females may encounter potential mates and how this may influence the results collected is evidently important. Future studies of mate choice should aim to mimic such conditions in the lab, to approximate what is occurring in actual arenas of mate choice. One limitation of this experiment is that all females were tested with the simultaneous comparison first and the sequential comparison second. Whilst there were no order effects on female preference in this study or a previous study on female green swordtails (Caves and Kelley 2023), we cannot fully exclude the possibility that order effects may have affected the results.

## Conclusion

Whilst female swordtails preferred larger males, and had a stronger preference when these males were presented simultaneously rather than sequentially, it is unclear what cognitive mechanisms they use to compare male stimuli over time. Although previous studies have shown that this species, amongst others, uses proportional processing to compare mate choice stimuli, we were only able to demonstrate that in this experiment that females compared both current and previous males when making a mate choice decision during sequential presentation of mates, but not show a specific mechanism. This highlights just how variable mate choice can be, even under laboratory conditions. Future studies should aim to further investigate these mechanisms in other species, taking into account simultaneous vs. sequential experimental design, in order to disentangle how widespread proportional processing is in mate choice, and what factors may increase variability in results. It would also be interesting to investigate further into the stochastic decision-making of how female swordtails specifically use previously seen males or previous mates to dictate their preferences, and how far back in time they may use this information.

## Supplementary Material

araf108_Supplementary_Data

## Data Availability

Analyses reported in this article can be reproduced using the data provided on Dryad by Bullough et al. (2025).
